# “Vascular inflammation and cardiovascular disease: review about the role of PET imaging”

**DOI:** 10.1007/s10554-022-02730-9

**Published:** 2022-10-18

**Authors:** Antonio Maria Sammartino, Raffaele Falco, Andrea Drera, Francesco Dondi, Pietro Bellini, Francesco Bertagna, Enrico Vizzardi

**Affiliations:** 1 Institute of Cardiology, Department of Medical and Surgical Specialties, Radiological Sciences and Public Health, ASST Spedali Civili, University of Brescia, Brescia, Italy; 2 Nuclear Medicine, Department of Medical and Surgical Specialties, Radiological Sciences and Public Health, ASST Spedali Civili Di Brescia, University of Brescia, Brescia, Italy

**Keywords:** ^18^F-FDG, ^18^F-FNa, PET, Vascular inflammation, Cardiovascular disease (CVD), Atherosclerosis

## Abstract

Inflammation characterizes all stages of atherothrombosis and provides a critical pathophysiological link between plaque formation and its acute rupture, leading to coronary occlusion and heart attack. In the last 20 years the possibility of quantifying the degree of inflammation of atherosclerotic plaques and, therefore, also of vascular inflammation aroused much interest. ^18^Fluoro-deoxy-glucose photon-emissions-tomography (^18^F-FDG-PET) is widely used in oncology for staging and searching metastases; in cardiology, the absorption of ^18^F-FDG into the arterial wall was observed for the first time incidentally in the aorta of patients undergoing PET imaging for cancer staging. PET/CT imaging with ^18^F-FDG and ^18^F-sodium fluoride (^18^F-NaF) has been shown to assess atherosclerotic disease in its molecular phase, when the process may still be reversible. This approach has several limitations in the clinical practice, due to lack of prospective data to justify their use routinely, but it’s desirable to develop further scientific evidence to confirm this technique to detect high-risk patients for cardiovascular events.

## Introduction

Current evidence has shown a central role for inflammation in all stages of the atherosclerotic process.

Numerous studies have identified how inflammation is widely involved in early atherogenesis, in progression of lesions and in thrombotic complications of the disease and how circulating markers of inflammation and the propensity to develop ischemic events are strongly related [1].

**Fig. 1:**
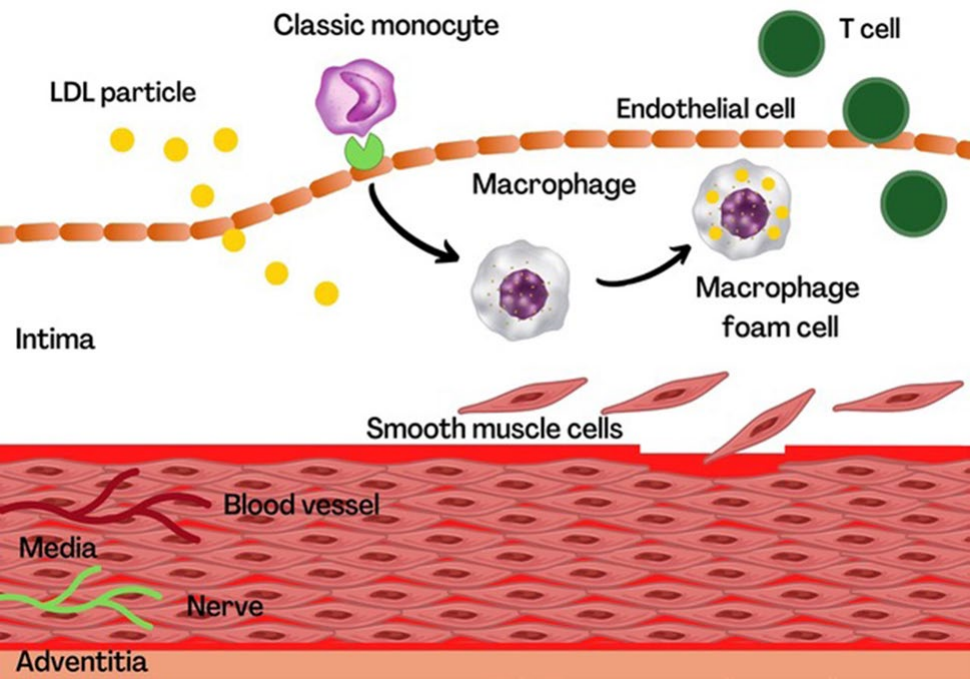
Initiation of atherosclerotic plaque. *LDL LDL-cholesterol; *SMC* smooth muscle cells; *T cell* T-lymphocyte. (Adapted by Libby P, Buring J.E, Badimon L, et al*.* Atherosclerosis. *Nature Reviews Disease Primers* (2019) 5; 56.)

Inflammation characterizes all phases of atherothrombosis and provides a critical pathophysiological link between plaque formation and its acute rupture, which leads to coronary occlusion and heart attack. High levels of cholesterol and triglycerides in the blood cause the small particles of lipoproteins to bind to proteoglycans and accumulate in the intima layer; the lipoprotein particles bound to proteoglycans have a greater susceptibility to oxidation (Fig. 1). Oxidative stress, including products present in modified lipoproteins, induces the expression of cytokines and chemokines which, locally, recall leukocytes.

**Fig. 2:**
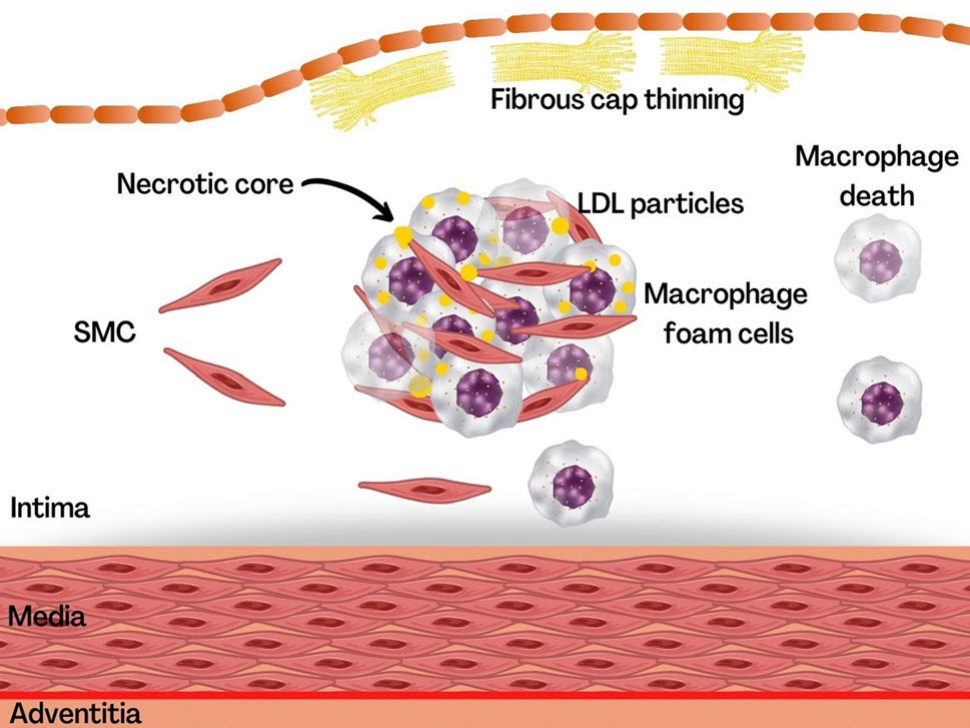
Progression plaque and fibrous cap. *LDL LDL-cholesterol; *SMC* smooth muscle cells; *T cell* T-lymphocyte. (Adapted by Libby P, Buring J.E, Badimon L, et al*.* Atherosclerosis. *Nature Reviews Disease Primers* (2019) 5; 56.)

In response to chemotactic cytokines (e.g., Monocyte Chemotactic Protein-1 [MCP-1]), monocytes cross the artery wall and amplify the release of growth factors and stimulants for formation of macrophages. As the process progresses, foam cells develop, which are macrophages filled with oxidized lipoproteins and fats. The cascade of local chemokines continues until it attracts smooth muscle cells, which begin to process the extracellular matrix and the “fatty strip” evolves into a fibro-adipose lesion. In the later stages calcification can occur (Fig. 2), first as microcalcification of the vessels, then as macrocalcification last to a formation of a core [2–5].

To date, therefore, it is well known that vascular inflammation is a central component of the atherosclerotic process as supported by several experimental studies, observational data and by demonstration of potential beneficial effects of anti-inflammatory therapies in advanced atherosclerotic disease [6].

Often cardiovascular clinical events, such as myocardial infarction, result from sudden rupture of atherosclerotic plaques and inflammation and plaque erosion are the main factors [7].

### Evolution of an atherosclerotic plaque

#### Positron Emission Tomography (PET) and cardiovascular diseases

The PET method has a relatively low spatial resolution [3–4 mm], which requires the use of simultaneous structural imaging (CT or MRI) to guide the localization of the ^18^F-fluorodeoxyglucose (^18^F-FDG) signal.

Widely used in oncology, for staging and searching metastases, in cardiology, ^18^F-FDG-PET is commonly used to estimate the consumption of glucose in the myocardium and therefore evaluate the residual vitality and possible benefit to myocardial revascularization.

Since several years, ^18^F-FDG-PET is widely used in case of suspicious of endocarditis or with suspected cardiac implantable electronic device (CIED) infection or LVAD (Left Ventricular Assistance Devices) infections. The sensitivity and specificity of [^18^F]FDG PET/CT in prosthetic valve endocarditis are 73–100% and 71–100%, respectively. [^18^F]FDG PET/CT also improved the sensitivity of the modified Duke criteria from 52–70% to 91–97% _(8)._

In native valve endocarditis, [^18^F]-FDG PET/CT has a relatively limited role, but it has a potential usefulness to identify extracardiac manifestations (ie, embolic stroke or septic embolization) [9].

[^18^F]FDG PET/CT shows very high diagnostic accuracy in detecting pocket/generator infection (sensitivity = 93%, specificity = 98%) and in cases of lead-related IE, [^18^F]-FDG PET/CT is highly specific (88%) with low sensitivity (65%), due to small vegetation(s) characterized by low-metabolic activity [10, 11].

Moreover, FDG-PET has emerged as the most commonly used technique to assess the extension of systemic sarcoidosis and also to assess extent and activity of myocardial involvement. In addition, FDG-PET in conjunction with MPI are recommended radionuclide method for evaluation of cardiac sarcoidosis, as well as to identify perfusion defects as important prognostic factor [12–14].

Furthermore, FDG PET/CT emerged as a useful modality for the diagnosis of large-vessel vasculitis, include giant cell arteritis and Takayasu arteritis, and evidence suggests that it provides independent prognostic information, as well as it can be used to monitor and modulate therapy [15, 16].

In recent years, as part of the expansion of imaging towards the identification of vascular inflammation, PET has been used to assessment of atherosclerotic disease at an early stage. In this field, ^18^F-FDG and ^18^F-sodium fluoride (^18^F-NaF) are the most commonly PET tracers used. ^18^F-FDG is absorbed by macrophages activated in plaques, highly sensitive for the metabolically active processes using glucose, while ^18^F-NaF is deposited in the microcalcification sites by chemical exchange of the ^18^F-ion with the hydroxyl group in the hydroxyapatite. PET/CT imaging with ^18^F-FDG and ^18^F-NaF has the ability to assess atherosclerotic disease in its molecular stage when the process may still be reversible [17–20].

#### The role of imaging in the atherosclerosis disease

In the last twenty years, the possibility of quantifying the degree of inflammation of atherosclerotic plaques and vascular inflammation raised a lot of interest. Traditional imaging modalities, such as ultrasound, computed tomography (CT), and magnetic resonance angiography (MRI), are all widely used in clinical practice to identify symptomatic macroscopic plaques, but have significant limitations to finding early stages of atherosclerosis when plaques are more biologically active [21, 22].

These evidences have led to expand imaging beyond the traditional anatomical and physiological domains, exploiting the metabolic processes underlying cardiovascular diseases [23], and to identify diagnostic methods capable to detecting atherosclerosis before the incidence of cardiovascular disease and in early stages of the disease [24, 25].

##### ^*18*^*F-FDG and PET*

During inflammatory processes, in a molecular point of view, cells must use exogenous glucose as fuel and since ^18^F-FDG is an analogue of glucose, it absorbed by macrophages resident in plaque and making them detectable by PET imaging [26, 27].

As it illustrated before, atherosclerotic plaques are rich in macrophages and other inflammatory cells and Tawakol et al*.* were able to demonstrate that the most inflamed areas of plaque accumulate almost 20 times more ^18^F-FDG than the control arteries. They confirmed that the metabolic signal measured in the plaques is mainly due to inflammatory activity and to the presence of macrophages [28, 29].

In addition to the macrophage content, there are also other circulating inflammatory biomarkers that determine a relationship between arterial ^18^F-FDG signal and inflammation, such as for example C-reactive protein (PCR), interleukin-6 (IL-6), selectin -P-soluble, selectin-E-soluble [30, 31] and these data confirm relationship between inflammatory process and progression of atherosclerosis disease.

##### ^*18*^*F-NaF and PET*

Over the past decade, interest in ^18^F-NaF has been revived by its ability to detect molecular calcifications in plaques by being absorbed only at sites of active calcification/ossification and in no other organ or pathological process [32].

Furthermore, ^18^F-NaF is rapidly cleared from the bloodstream (60–90 min) reaching a high contrast between the calcification sites and background activity [33, 34]_._

The uptake of ^18^F-NaF from the arterial wall is more consistently related to cardiovascular risk factors than the accumulation of ^18^F-FDG [35–36].

In fact, it has been shown that ^18^F-NaF deposits in the arterial level are more consistent in patients with angina than in healthy control subjects [37, 38]. Furthermore, coronary uptake of ^18^F-NaF has been shown to precede gross calcification visible on intravascular or CT ultrasound and ^18^F-NaF does not accumulate in the myocardium, contrary to high physiological myocardial uptake of ^18^F-FDG [39, 40].

#### Prognostic role of PET imaging

In a rabbit atherosclerosis model, authors observed when plaque rupture is promoted by venom injection, only aortic plaques with the highest pre-rupture ^18^F-FDG levels progress and undergo rupture and thrombosis [41].

These finds led to speculation a possible predictive role of ^18^F-FDG imaging for plaque rupture events and, therefore, major cardiovascular events (e.g., heart attack).

In vivo, some evidences correlated arterial absorption of ^18^F-FDG with subsequent risk of both plaque rupture and clinical events. Patients with the highest uptake of ^18^F-FDG were more likely to have previously had a vascular event or to have experienced one during the 6 months following PET imaging [42].

Moreover, Arauz et al*.* presented prognostic data in patients with symptomatic carotid artery disease; 85% of subjects had high absorption levels of ^18^F-FDG and they had worse outcomes over following 6 months (recurrent stroke, stent artery death or restenosis) than those with initially lower ^18^F-FDG levels [43].

More recently, McCabe et al*.* provided that in individuals with recent ischemic stroke/TIA and ipsilateral carotid stenosis, carotid plaque inflammation-related ^18^FDG uptake on PET/CT angiography was associated with 5-year recurrent ipsilateral stroke [44].

In last years, numerous studies were performed using ^18^F-FDG-PET to assess vascular inflammation in different subset of patients. In a large cohort of cancer patients (932 patients), asymptomatic for cardiovascular diseases, it was demonstrated that arterial uptake of ^18^F-FDG and calcifications in large arteries is related to the highest risk for a future vascular event (ischemic stroke, myocardial infarction, need for revascularization) [45].

In other smaller studies were found a significant relationship between the absorption of ^18^F-FDG in the arterial wall and the degree of inflammation and atherosclerosis plaques, respectively in end-stage renal disease (ESRD) patients and in chronic obstructive pulmonary disease (COPD) [46–48]. They confirmed how well-known conditions (COPD and ESRD) characterized by systemic inflammation are related to a higher grade of vascular inflammation and subclinical atherosclerosis and so an augmented risk for cardiovascular events.

Joshi AA et al. reached to the same conclusion demonstrating how the presence of aortic vascular inflammation detected by PET/CT with ^18^F-FDG is associated with the presence of coronary artery disease assessed by coronary CT (CCT) [49]_._

In a cross-sectional cohort study (215 patients) affected by psoriasis, authors quantified aortic vascular inflammation using ^18^F-FDG-PET/CT and at the same time they assessed the degree of coronary artery disease with CCT. They showed that extension of CAD (as higher prevalence of luminal stenosis, more severe luminal stenosis and higher prevalence of high-risk plaque) is greater in subjects with high aortic vascular inflammation and, regardless of cardiovascular risk factors, a strong association between aortic vascular inflammation and CAD was found.

This helped to demonstrate how aortic vascular inflammation assessed by ^18^F-FDG-PET/CT can be a potential surrogate for the assessment of early CAD.

#### PET imaging to evaluate anti-inflammatory therapies response

In last two decades, the grade of inflammation by PET/CT imaging as a surrogate marker of inflammatory activity in atherosclerosis has also been used for observing and evaluating the response to therapies [50, 51].

CANTOS [52] and COLCOT [53] trials demonstrated respectively that canakinumab (monoclonal antibody targeting interleukin-1β) and colchicine led to a significantly lower rate and lower risk of cardiovascular events. They proved that therapies targeting inflammation or immune-system pathway can improve cardiovascular outcomes.

According with these evidences, a single-center open-label study for first [54] and few years later a multicenter trial [55] observed a significant dose response in the reduction in FDG uptake between the high- and low-dose statin groups and showed that an incremental benefit of statin intensification was related to a lower plaque inflammation measured using FDG-PET imaging. In line with observations from large-scale trials of low-versus high-dose statins [56, 57], these data support the hypothesis that the cardiovascular benefit from statin therapy may be due to a rapid reduction in arterial inflammation.

In this context, PET imaging can provide a direct measure of inflammation arising in the vascular wall and it will also support early assessment of treatment effect of anti-inflammatory drugs [58, 59], as well as it will useful to evaluate the benefit of non-immunomodulator drugs in the inflammation processes [60].

#### ^*18*^*F-FDG or 18F-NaF in coronary heart disease*

Several technical hurdles must be overcome before ^18^F-FDG PET imaging can be applied to measure inflammation at the coronary plaque level. The significant uptake of ^18^F-FDG into the myocardium, tissue with physiological increased glucose metabolism, has prevented the use of this technology to detect atherosclerosis in coronary arteries, a major cause of population morbidity and mortality in the Western world [61].

Cardiac motion also leads to blurring of coronary plaque uptake of ^18^F-FDG, especially in the distal coronary arteries. Routine PET acquisitions are typically gated and obtained in 15 min, therefore, there may be spatial discordance between PET and CT images. The small coronary plaque size also requires a relatively high degree of focal ^18^F-FDG accumulation before a detectable signal can be measured due to modest resolution of PET. The main challenge seems to relate to the combined effects of heart and respiratory movements during data acquisition over a long period of time, as well as the difficulty in identifying small plaques (1–2 mm) using the tracers [62].

Furthermore, mechanisms other than inflammation can generate the ^18^F-FDG signal associated with atheromas. An example, hypoxia can lead to an increase in the use of glucose by macrophages; in fact, hypoxia stimulates glucose intake by the cells and, like inflammation, can cause an increased absorption of ^18^F-FDG in the cells present in atheromas [63].

Despite these challenges and limitations, several working groups have investigated the accumulation of ^18^F-FDG on coronary vessels [64, 65].

Particularly, comparing uptake of ^18^F-FDG at the level of coronary arteries with culprit lesions in patients with acute coronary syndrome (ACS) and stable angina controls, Rogers et al.they observed a significantly higher uptake of ^18^F-FDG from the coronary artery, higher in plaques of ACS patients. This finding is consistent with the notion that SCA is associated with increased arterial inflammation [66].

Although ^18^F-FDG PET has been shown to be useful for quantifying inflammation within atherosclerosis, Joshi et al. [67] were among the first to demonstrate the superiority of ^18^F-NaF PET-CT as the first non-invasive imaging method to identify and locate ruptured or high-risk coronary plaque for rupture. In their prospective clinical study, patients with myocardial infarction (n = 40) and stable angina (n = 40) who underwent PET-CT with ^18^F-NaF and 18F-FDG and coronary angiography were enrolled. It has been shown that intense absorption of 18F-NaF is localized at the level of the plaque undergoing recent rupture in patients with acute myocardial infarction. The fact that 18F-NaF can also be used to detect plaque in coronary arteries is a big plus for this tracer [68, 69].

In recent years, a major trial (CAMONA trial: Cardiovascular Molecular Calcification Assessed by 18F-NaF PET/CT) has been conducted to compare the performance of ^18^F-FDG- and ^18^F-NaF-PET in atherosclerosis.

Healthy volunteers and patients with a previous episode of chest pain was recruited to study the relationship between CVD risk, estimated by the Framingham Risk Score (FRS) and arterial inflammation. The study found that the increased risk of CVD is associated with marked increases in vascular calcification, assessed by PET imaging with ^18^F-NaF, and by vascular calcium load, assessed by imaging CT scan, but not associated with the degree of arterial inflammation, evaluated by ^18^F-FDG PET imaging [70, 71].

#### Outlook: new nuclear probes for vascular inflammation and atherosclerosis

Alternative molecular targets and PET tracers for imaging vascular inflammation are being studied and, according pathophysiology of the inflammation, their target is not directly linked to inflammation but it’s also direct to plaques microcalcifications, hypoxia or apoptosis process. Immune and inflammation cells are associated with cytokine and receptors expression, resulting in a numerous potential imaging targets for new PET tracers (Table 1).

**Table 1:** Registered ongoing clinical trials evaluating nuclear probes for vascular inflammation in atherosclerosis

Molecular target	Nuclear probes	Mechanism target	Clinical trials
GLUT transporters	[^18^F]FDG	Macrophage metabolism	NCT04181996
			NCT00633022
			NCT01341730
			NCT01186666
			NCT02162303
			NCT03215550
			NCT04505865
			NCT04350216
Choline transporter	[^18^F]FMCH	Macrophage activity	NCT03252990
			NCT02640313
SSTR2	[^68^ Ga]Ga-DOTA-TATE	Macrophage activity	NCT04043377
			NCT04073810
			NCT02021188
Mannose receptors	[^68^ Ga]Ga-NOTA-MSA	Macrophage activity	NCT01893489
	[^99m^Tc]Tc-Tilmanocept		NCT01889693
	[^68^ Ga]Ga-NOTA-anti-MMR-VHH2		NCT02542371
			NCT04758650
TSPO	[^11^C]PBR28	Macrophage activity	NCT00547976
	[^11^C]PK11195		
Integrins	[^18^F]RGD-K5	Neoangiogenesis and macrophage activity	NCT03364270
	[^68^ Ga]Ga-NOTA-RGD		
FAP	[^68^ Ga]Ga-DOTA-FAPI-04	Proinflammatory macrophages and type I collagen breakdown in fibrous caps	NCT05036759
CCR2	[^64^Cu]Cu-DOTA-ECL1i	Pro-inflammatory macrophages	NCT04537403
Aβ	[^18^F]flutemetamol	Aβ deposition in human atherosclerotic plaques	NCT03291093
NPR-C	[^64^Cu]Cu-DOTA-CANF-Comb	Endothelial and vascular smooth muscle cells activation	NCT02498379
			NCT02417688
–	[^64^Cu]Cu-macrin	Macrophage phagocytic activity	NCT04843891

[^*18*^*F-FDG* 2-[18F]fluoro-2-deoxy-d-glucose, *SSTR2* somatosatin receptor 2, *68 Ga* Gallium-68, *64Cu* Copper-64, *[18F]FMCH* [18F]fluoromethylcholine, *[68 Ga]Ga-NOTA-MSA* [68 Ga]Ga-NOTA-neomannosylated human serum albumin, *anti-MMR-VHH2* nanobody targeting macrophage mannose receptor, *[11C]PBR28* [11C]N-acetyl-N-(2-methoxybenzyl)-2-phenoxy-5-pyridinamine), *[18F]RGD-K5* 18F-fluorination of an arginine-glycine-aspartic acid derivated peptide targeting integrin αvβ3, *FAP* fibroblast activating protein, *FAPI* fibroblast activating protein inhibitor, *CCR2* C–C chemokine receptor 2, *ECL1i* extracellular loop 1 inverso, *Aβ* beta amyloid deposits. [Table adapted from Prigent *et* Vigne (76)]

Moreover, thanks to development of nanotechnologies, nanoparticles are emerging to detect and quantify macrophages and atherosclerotic plaques. They have the great advantage to be easily tunable and often they present longer half-lives than others PET radionuclides [72–75], but more data and prospective study in human are needed.

A great review about PET tracers in vascular inflammation, with a focused update on recent radiopharmaceuticals research related to nuclear imaging and an overview of ongoing research in this field, it’s been recently done by Prigent et Vigne [76].

It desirable that the development of PET probes will implement nuclear traces able to explore new molecular targets and PET imaging allows for direct visualization of metabolic processes, including other inflammation mechanisms unknown yet.

#### Future prospects for PET imaging

As emerged from this review, PET imaging could either be used to further stratify groups of high-risk patients and evaluate therapies response.

However, although a link between vascular inflammation and future cardiovascular risk identified by ^18^F-FDG PET can be implied through its association with clinical risk factors, inflammatory biomarkers, high-risk plaque, stroke recurrence and major adverse clinical events in retrospective analyses of large PET imaging datasets, definitive prospective clinical outcome data is needed. In the near future ^18^F-NaF could be play a key role to detect atherosclerosis, but considering the limited data available, it is certainly desirable to develop further scientific evidence to confirm this technique and new PET tracers as a good weapon to intercept high-risk patient and prevent major cardiovascular events.
